# Phenotypic expression and clinical outcomes in a South Asian PRKAG2 cardiomyopathy cohort

**DOI:** 10.1038/s41598-020-77124-9

**Published:** 2020-11-26

**Authors:** Hisham Ahamed, Aniketh Vijay Balegadde, Shilpa Menon, Ramesh Menon, Aishwarya Ramachandran, Navin Mathew, K. U. Natarajan, Indu Ramachandran Nair, Rajesh Kannan, Meghna Shankar, Oommen K. Mathew, Thong T. Nguyen, Ravi Gupta, Eric W. Stawiski, V. L. Ramprasad, Somasekar Seshagiri, Sameer Phalke

**Affiliations:** 1 Amrita Institute of Medical Sciences and Research, Kochi, India; 2 MedGenome Labs, Bangalore, India; 3 AgriGenome Labs, Kochi, India; 4 Genentech Inc., South San Francisco, USA; 5 MedGenome Inc., Foster City, USA; 6 SciGenom Research Foundation, Kochi, India; 7 SciGenom Labs Pvt Ltd, Kochi, India

**Keywords:** Clinical genetics, Cardiology, Cardiomyopathies

## Abstract

The PRKAG2 syndrome is a rare autosomal dominant phenocopy of sarcomeric hypertrophic cardiomyopathy (HCM), characterized by ventricular pre-excitation, progressive conduction system disease and left ventricular hypertrophy. This study describes the phenotype, genotype and clinical outcomes of a South-Asian PRKAG2 cardiomyopathy cohort over a 7-year period. Clinical, electrocardiographic, echocardiographic, and cardiac MRI data from 22 individuals with PRKAG2 variants (68% men; mean age 39.5 ± 18.1 years), identified at our HCM centre were studied prospectively. At initial evaluation, all of the patients were in NYHA functional class I or II. The maximum left ventricular wall thickness was 22.9 ± 8.7 mm and left ventricular ejection fraction was 53.4 ± 6.6%. Left ventricular hypertrophy was present in 19 individuals (86%) at baseline. 17 patients had an WPW pattern (77%). After a mean follow-up period of 7 years, 2 patients had undergone accessory pathway ablation, 8 patients (36%) underwent permanent pacemaker implantation (atrio-ventricular blocks—5; sinus node disease—2), 3 patients developed atrial fibrillation, 11 patients (50%) developed progressive worsening in NYHA functional class, and 6 patients (27%) experienced sudden cardiac death or equivalent. PRKAG2 cardiomyopathy must be considered in patients with HCM and progressive conduction system disease.

## Introduction

Hypertrophic cardiomyopathy (HCM) is the most common genetic cause of cardiomyopathy worldwide and a genetic origin for this heterogenous group of diseases is found in ~ 40–60% of HCM patients, usually with an autosomal dominant mode of inheritance^1,2^. It is characterized by a hypertrophied, non-dilated left ventricle, without evidence of any other cardiac or systemic disease, leading to heart failure and sudden cardiac death (SCD). The mutations responsible for HCM are often localized to genes encoding for sarcomeric proteins^3^. There are several other genetic cardiomyopathies which are not caused by cardiac sarcomere mutations and yet they share many of the phenotypic manifestations of HCM such as evidence of left ventricular hypertrophy on the electrocardiogram and echocardiogram. These HCM phenocopies include lysosomal storage disorders, glycogen storage disorders, mitochondrial cytopathies, fatty acid metabolism disorders and cardiac amyloidosis^4^. PRKAG2 (Protein Kinase Adenosine monophosphate activated Gamma 2 non-catalytic subunit 2) cardiomyopathy, an important HCM phenocopy, is a rare autosomal dominant, non-lysosomal glycogen storage disease characterized by ventricular pre-excitation, supra-ventricular arrhythmias and cardiac hypertrophy. The clinical phenotypes of PRKAG2 cardiomyopathies often overlap with HCM due to sarcomere protein mutations and often leads to misdiagnosis^5^.

In 2001 PRKAG2 cardiomyopathy was found to be caused by mutations in the gene encoding ɣ2 regulatory subunit (PRKAG2)^6^ of the 5′ AMP activated protein kinase (AMPK). AMPK is a highly conserved, ubiquitously expressed serine/threonine protein kinase responsible for cellular energetic homeostasis control. γ2 regulatory subunit of AMPK (PRKAG2) binds AMP, enhancing the activation of the catalytic α-subunit^7,8^. *PRKAG2* mutations are suspected to modify the three-dimensional structure of AMPK, altering its affinity for AMP and modifying the enzyme activity which alters the myocyte glucidic uptake and metabolism causing the deposition of glycogen. These glycogen-filled myocytes interfere with the normal atrio-ventricular septation and leads to the observed cardiac phenotype.

Overall about 24 pathogenic *PRKAG2* mutations^9^ have been identified showing clear genotype/phenotype correlations in PRKAG2 cardiomyopathies. The actual prevalence of PRKAG2 cardiomyopathies has not been properly investigated and only about 200 cases have been reported worldwide so far. Different studies in the US and Europe on large populations have given different estimates. There are no studies published on PRKAG2 cardiomyopathies from the South Asian region.

Here, we report the morphological expression and clinical course of a cohort of 22 PRKAG2 cardiomyopathy patients belonging to three unrelated families, identified at the Amrita HCM center. The quantum of literature on PRKAG2 cardiomyopathy and its outcomes have been limited and hence, this 7.0 ± 1.5 year follow up data aims to throw light on the distinctive clinical features, natural history and outcomes of this disease.

## Methods

### Study cohort

This study was performed at the Amrita HCM center at the Amrita Institute of Medical Sciences and Research in accordance with ICMR National Ethical Guidelines for Biomedical and Health Research. All aspects of the study were approved by Amrita Institutional Ethics Committee. The families were recruited in the framework of Familial Genetic Disorder Study (FGDS) program by MedGenome from the Amrita HCM center registry which, at the time of writing, consisted of 1640 patients with a clinical diagnosis of HCM. An informed consent was obtained from all the subjects or from a parent and/or legal guardian if subjects were under 18 years of age. The cohort consisted of three unrelated families, Family A (FGD0128; n = 31), Family B (FGD0137; n = 9) and Family C (FGD0314; n = 10) from Ernakulam and Trichur district in Central Kerala, India. At least more than one individual in each of these families were diagnosed with HCM. The proband belonging to each family and all the family members considered to be at risk (Ages 7–64; Mean 39.5 ± 17.6 years) were investigated.

### Clinical analysis

Clinical analysis involved review of electronic hospital records, 12—lead electrocardiography, echocardiography, 24-h ambulatory Holter monitoring and cardiac MRI. Endomyocardial biopsy was performed in selected patients according to standard methodology and the tissue sections were stained with hematoxylin and eosin and per-iodic acid Schiff stain.

The clinical phenotype of the PRKAG2 cardiac syndrome was based on cardiac hypertrophy, pre-excitation pattern on the ECG and evidence of conduction system disease (either on the surface ECG or electrophysiological study). Cardiac hypertrophy reflecting HCM was considered to be present if there was unexplained maximal wall thickness > 15 mm in any myocardial segment, septal/posterior wall thickness ratio > 1.3 in normotensive patients or septal/posterior wall thickness ratio > 1.5 in hypertensive patients. A short PR interval (< 120 ms), widened QRS duration (QRSd > 110 ms) and a delta wave (abnormal initial QRS vector) made up the criteria for ventricular pre-excitation (WPW pattern). Electrophysiologic studies demonstrating accessory pathways (anterogradely conducting) was also taken as a defining criterion for ventricular pre-excitation. Sinus node dysfunction or atrio-ventricular blocks on the surface electrocardiogram or electrophysiologic study was considered indicative of conduction system disease.

### DNA isolation, exome library preparation and sequencing

Genomic DNA was isolated from whole blood using QIAamp DNA Blood Mini Kit (Qiagen, Germany) and quantified using Qubit fluorometry (Thermo Fisher Scientific, USA). For library preparation, 200 ng of the Qubit quantified DNA was fragmented to 180–220 bp inserts. The fragments were then end-repaired, 3′ adenylated and ligated with the indexed adapters. The adapter-ligated fragments were then amplified with adapter-specific primers followed by size selection and purification to generate gDNA library. The gDNA library was then hybridized to xGen Exome Research Panel v1.0 (Biotin labelled baits, Integrated DNA Technologies) for 4 h at 65 °C. Post incubation, the hybridized libraries were captured using Streptavidin M270 beads (Thermo Fisher Scientific) followed by stringent washes to remove unbound DNA molecules. The enriched libraries were amplified and purified. The final library was assessed for fragment size distribution using Tape Station (Agilent, USA) and was quantified using Qubit (Thermo Fisher Scientific, USA) for Sequencing. The quantified libraries were sequenced on Illumina HiSeq 4000 to generate100bp paired end sequences.

### Data processing, variant calling and variant annotation

Quality check (QC) and adapter trimming for the sequenced reads were performed using fastq-mcf (version 1.04.676) (https://expressionanalysis.github.io/ea-utils/). The pre-processed reads were aligned to the reference human genome (hg19, Feb. 2009 release) downloaded from UCSC Database^10^ using BWA-mem^11^. Aligned read processing and variant calling steps were performed using Sentieon version of GATK^12^. Briefly, the aligned reads were sorted, and duplicate reads were removed. The reads were then realigned around the known indels from 1000G study downloaded from (ftp://ftp.broadinstitute.org/distribution/gsa/gatk_resources.tgz) for improving the accuracy of indel calls. Base quality score recalibration (BQSR) was done using known variants from dbSNP (v149). The germline variant calling was performed using Haplotyper command in Sentieon^12^.

The variants identified above were annotated using deep annotation VariMAT pipeline utilizing vep^13^ program and gene models downloaded from Ensembl database (release 84; ftp://ftp.ensembl.org/pub/release-84/gtf/homo_sapiens). The annotation pipeline included: annotation of variants into genes, repeats, genic features (exon, intron, 5′ UTR, 3′ UTR, coding-region, splice-site), transcripts, variant classes (silent, missense, non-sense, stop-loss, start-loss, in-frame, frameshift, etc.), known variants reported in public repositories (ExAC, gnomAD, 1000G, dbSNP, EVS, TwinsUK, JAP1000G, MedGenome-Germline Variant Database), pathogenicity classes based on known pathogenic variants (from ClinVar, HGMD, SwissVar), pathogenicity prediction programs (PolyPhen, SIFT, MutationTaster, MutationAssessor, LRT and others) and segregation of variants in the family. Identified variants were validated using Sanger sequencing.

### Kinship analysis

We have used the *–relatedness2* method of Vcftools (version 0.1.14) software for the Kinship analysis. The algorithm uses the method proposed by Manichaikul and colleagues^14^. The kinship coefficients matrix of 43 samples belonging to 3 families were subjected to hierarchical cluster analysis using MeV tool (version 4.9).

## Results

### Clinical features

**Figure 1:**
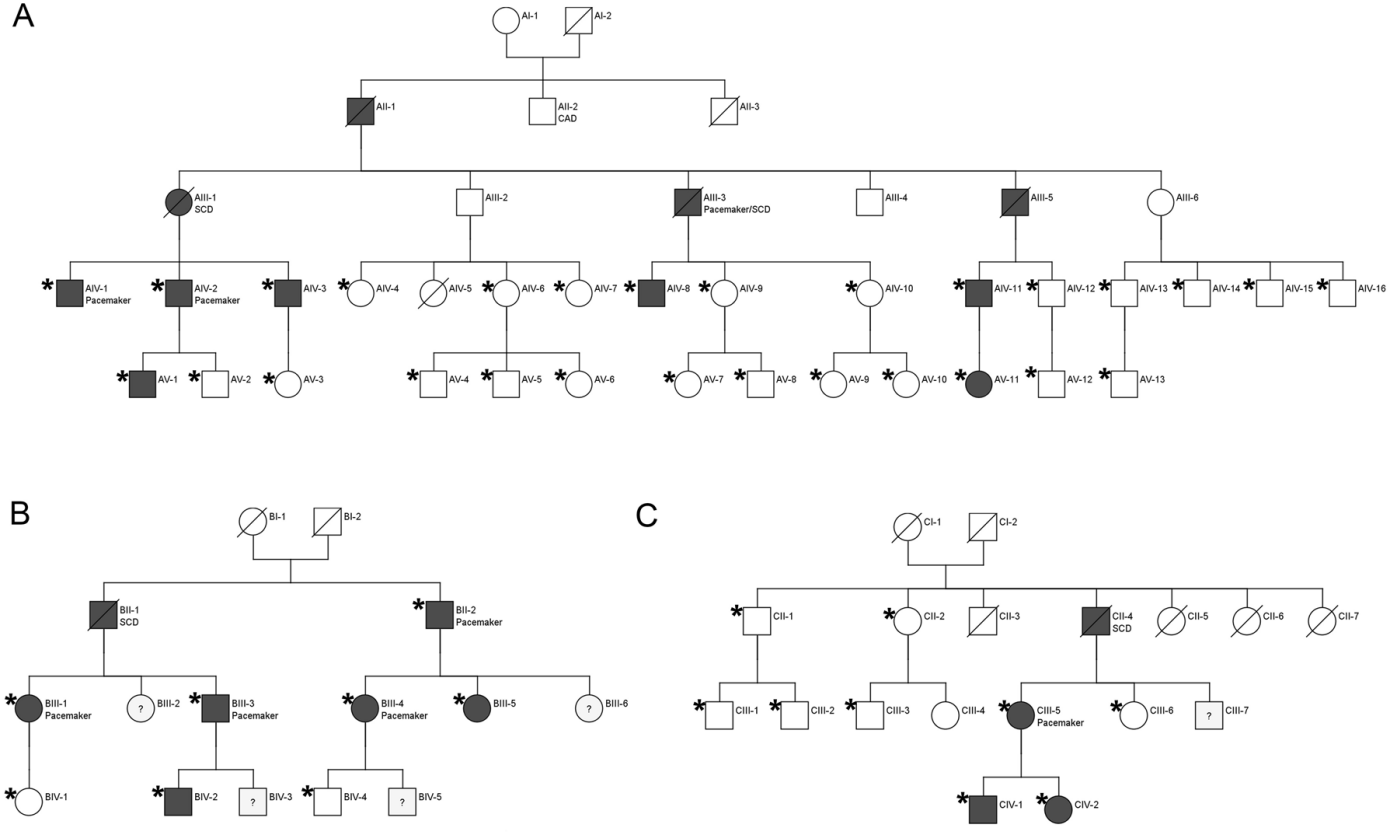
Detailed pedigree of the three families studied in this cohort. (**A**) Family A (FGD0128), (**B**) Family B (FGD0137) and (**C**) Family C (FGD0314). Individuals for whom whole exome sequencing was performed are highlighted with an asterisk (*). Square represents the males and the circles represent the females. Filled squares and the circles represent the affected individuals. Square or circles with question mark (?) represent the individuals with unknown phenotype. SCD represents the individual who had sudden cardiac death and “Pacemaker” represents the individuals who underwent pacemaker implantation.

Three index patients with the HCM phenotype from the three unrelated families (Family, A, Family B and Family C) and their at-risk family members (Fig. 1) were clinically and genetically evaluated. The clinical, demographic and cardiovascular outcome data have been summarized in Supplementary Table S1a, S1b and S1c. The study cohort comprised of 50 individuals, out of which, 22 individual patients with suspected PRKAG2 disease were identified (68% men; mean age 39.5 ± 18.1 years). The remaining 28 individuals did not exhibit the clinical phenotype of the disease.

At initial evaluation, 86% (19/22) of the 22 subjects (68% men; mean age 39.5 ± 18.1 years) had evidence consistent with PRKAG2 disease and were considered clinically affected while the remaining 3 individuals were without the definite clinical phenotype. The mean left ventricular maximum wall thickness (MWT) of the affected 19 patients was 25.3 ± 7 mm (MWT of 22 patients was 22.9 ± 8.7 mm). At cardiac diagnosis, all of the patients were in NYHA functional class I or II.

### Electrocardiogram

**Figure 2:**
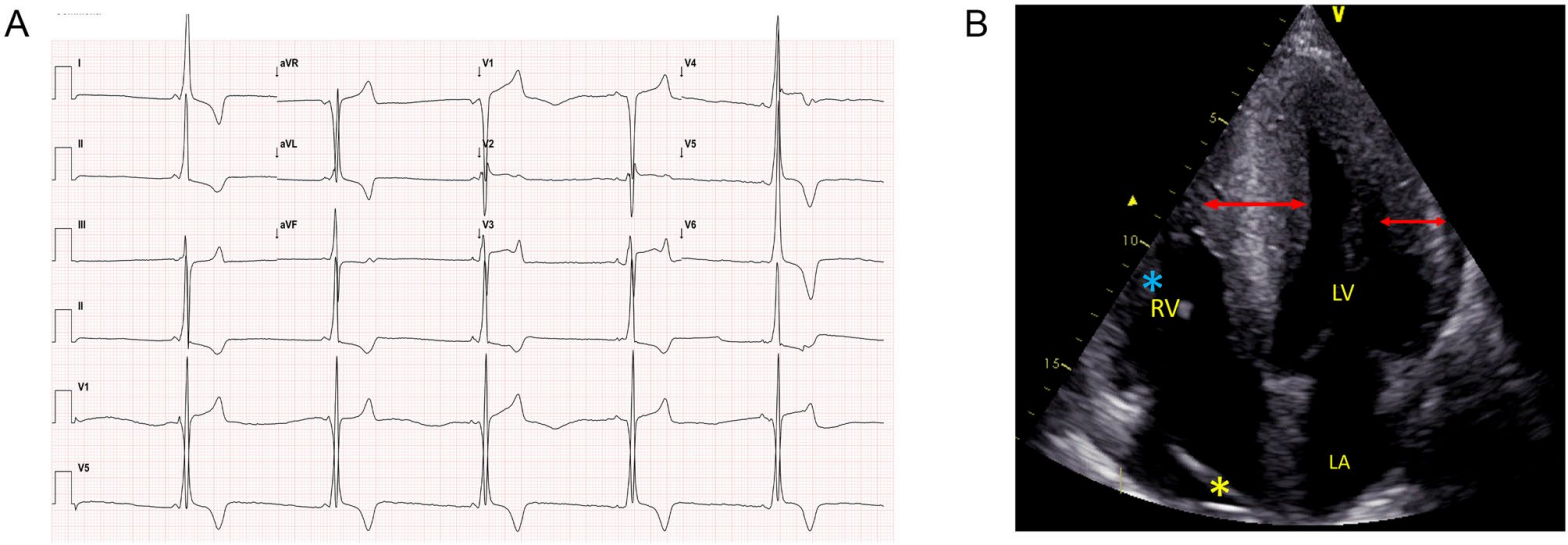
Typical example of an ECG and Echocardiogram of one of the patients with PRKAG2 cardiomyopathy. ECG of patient BIII-4 shows a short PR interval with a delta wave (WPW pre-excitation pattern) and markedly high voltages indicating left ventricular hypertrophy in the QRS complexes with deep T wave inversions (**A**). Transthoracic echocardiogram (apical four-chamber view) of patient AIV-2 showing marked hypertrophy of the left ventricle (IVS 28 mm; lateral wall 20 mm) along with right ventricular free wall hypertrophy (blue asterisk). Pacemaker lead visualized as highlighted by yellow asterisk (**B**). *IVS* Interventricular Septum

The electrocardiogram showed a WPW pattern in 17 patients (17/22; 77.2%). The electrocardiograms also showed markedly increased voltages for maximum R or S wave (25–70 mm; mean 34.9 ± 11.4 mm) and deeply inverted T-waves. Intraventricular conduction defects were noted in 6 patients. The mean PR interval duration for the 17 patients with a WPW pattern was 100 ± 9.5 ms while the mean PR interval for the entire 22 patient cohort was 104 ± 18 ms (ECG findings summarized in Supple Table S1a, S1b and S1c). A characteristic ECG of one of the patients is shown in Fig. 2A.

### Echocardiogram

Echocardiographic evaluation showed that 19 individuals (19/22;86%) had evidence of diffuse and concentric left ventricular hypertrophy (LVH). The mean left ventricular maximum wall thickness (N = 19) was 25.3 ± 6.5 mm (range 13–33 mm). The mean left ventricular maximum wall thickness for the entire 22 patient cohort was 22.9 ± 8.7 mm. Right ventricular hypertrophy was seen in 19 patients with LVH (19/22; 86%) with a mean right ventricular free wall thickness of 7.5 ± 1.3 mm (range 6–11 mm). The mean left ventricular ejection fraction of all the 22 patients was 53.4 ± 6.6% (40–65%). None of the 22 patients had left ventricular outflow tract obstruction > 30 mmHg or evidence of systolic anterior movement of the mitral valve. Key echocardiographic parameters are summarized in Supplementary Table S1a, S1b, S1c, S3a and S3b. A characteristic apical four chamber image of one of the patients is shown in Fig. 2B.

### Cardiac magnetic resonance imaging

**Figure 3:**
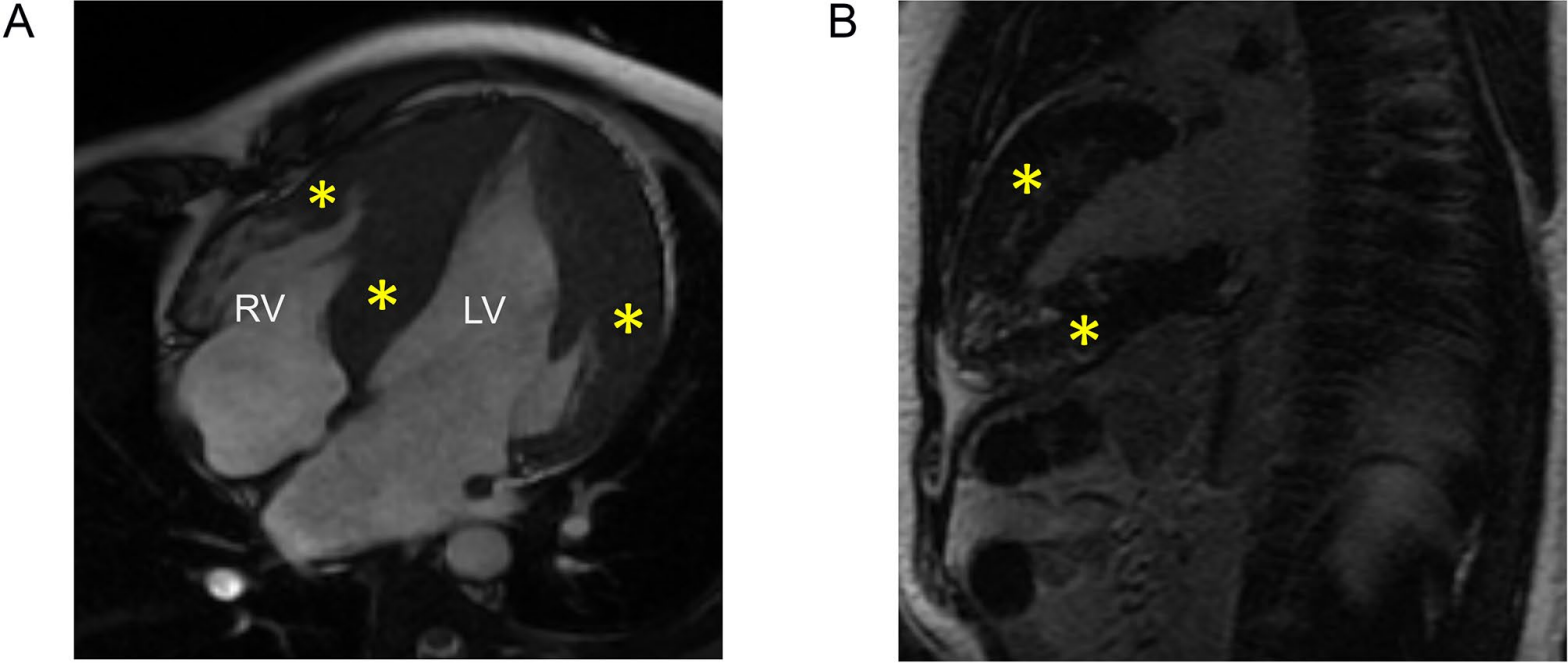
Cardiac MRI (SSFP Cine sequences) of patient CIII-5 showing marked hypertrophy in the region of the interventricular septum and lateral wall of the left ventricle (yellow asterisks) along with right ventricular free wall hypertrophy (red asterisk) in the apical four-chamber view (**A**). The two-chamber long-axis view shows patchy areas of mid-wall left ventricular late gadolinium enhancement in the anterior and inferior walls (yellow asterisks) (**B**).

To deep phenotype the *PRKAG2* cardiomyopathy patients, we performed cardiac MRI (CMR) on 8 subjects (8/22; 36% ) (AV-1, AIV-8, AIV-11, AV-11, BIV-2, CIII-5, CIV-1 and CIV-2) across three families. The studies showed diffuse and severe concentric LVH and right ventricular hypertrophy in the severely affected patients. Quantification of late gadolinium enhancement (LGE) in patients CIII-5 and BIV-2 were 10% and 9.7% respectively (Table 2). The remaining 6 patients with cardiac MRI studies did not have significant mid-myocardial or sub-endocardial enhancement on LGE studies (Fig. 3A,B). None of the patients showed LGE in the right ventricle. Cardiac MRI findings are summarized in Table 2.

### Endomyocardial biopsy

**Figure 4:**
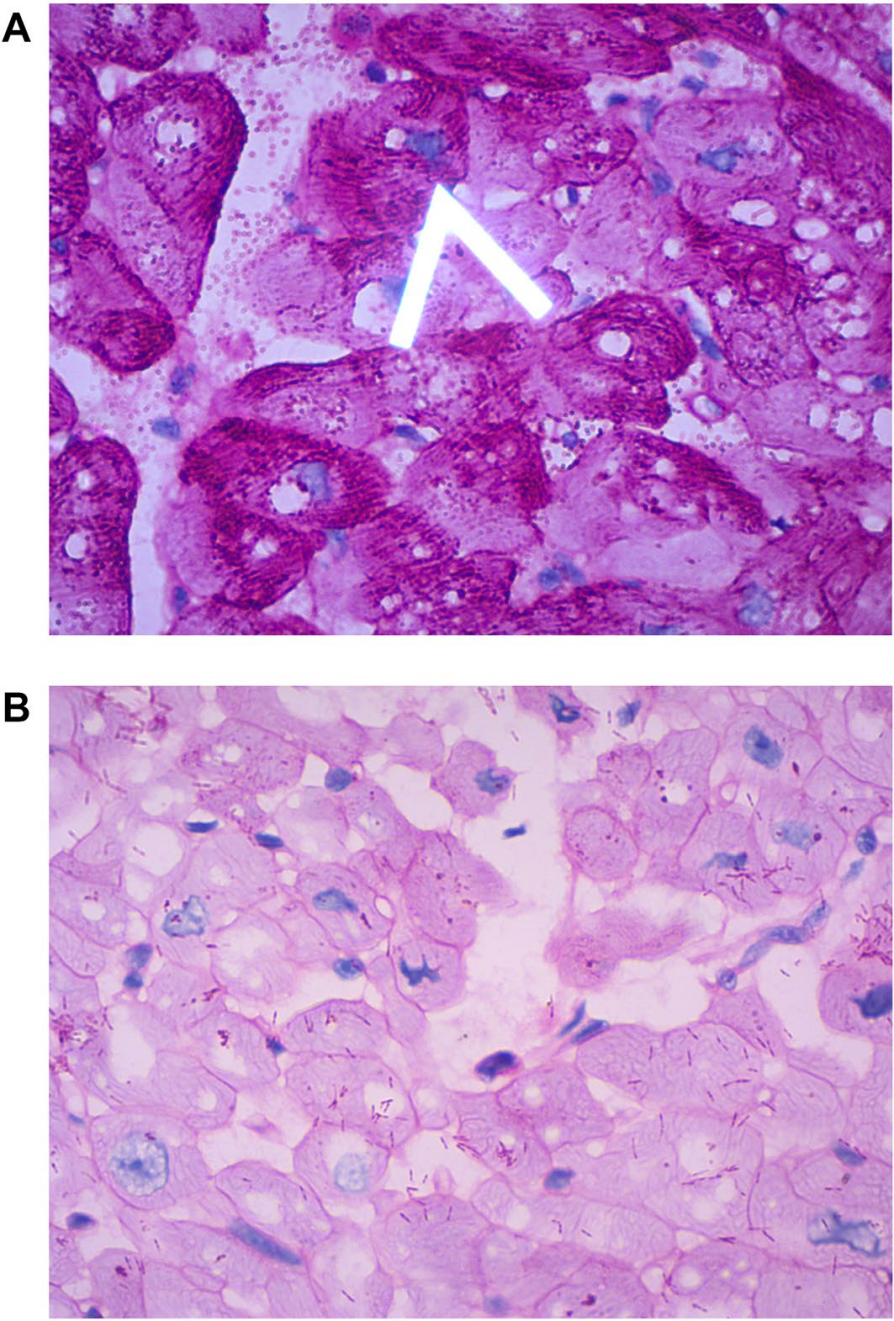
Endomyocardial biopsy with Periodic acid Schiff stain (PAS) showing abundant magenta positive granules (white arrow) in the cardiomyocyte cytoplasm indicating glycogen deposition. Enlarged cardiomyocytes were observed (**A**). Endomyocardial biopsy in Panel A treated with diastase (PAS-D) show marked clearing of the excessive glycogen deposition (**B**).

Endomyocardial biopsy of patient CIII-5 performed through the trans-jugular access, revealed predominantly enlarged myocytes (~ 200 microns), with nuclear enlargement. There was no significant myofibrillar disarray as seen in patients with HCM due to sarcomeric mutations. Per-iodic acid Schiff (PAS) staining revealed an abundance of magenta positive granules in the myocyte cytoplasm indicative of excessive glycogen deposition. When treated with diastase (PAS—D), there was marked clearing of the magenta positive granules containing glycogen (Fig. 4A,B).

### Exome sequencing and causal variant identification

In order to understand the causal mutation for the observed hypertrophic cardiomyopathy phenotype in the members of these 3 families we performed whole exome sequencing on 43 individuals out of 50 members screened clinically. Six members had sudden cardiac death (SCD) and their samples could not be included in the study. Whole exome capture panel was used for whole exome sequencing library preparation. An average exome coverage of 63 × (range 20–142×) with 91% of the bases over 20 × (range 76–91) coverage was obtained (Supplementary Table S2).

**Figure 5:**
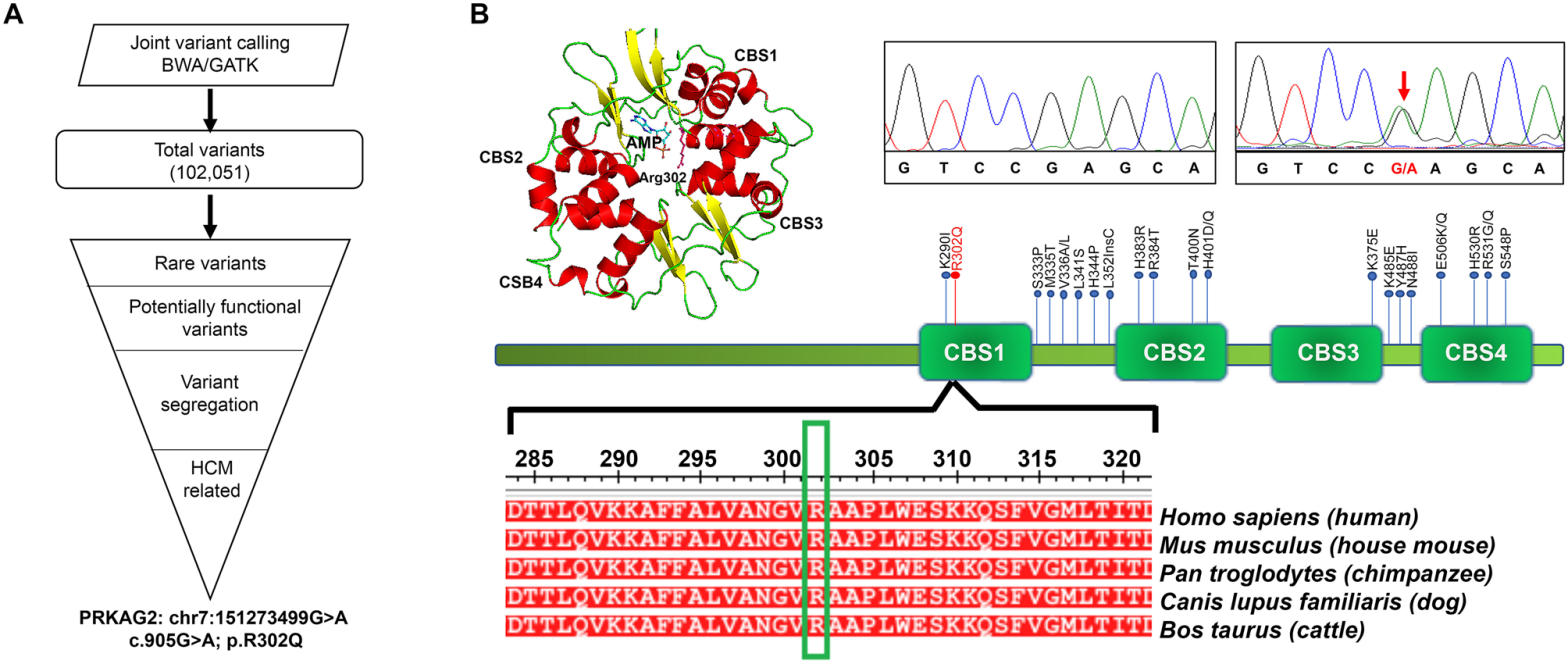
Identification of casual variants in the three families in the study cohort. Flowchart depicting the analysis of exome data from all the affected and unaffected members from the families. PRKAG2 c. 905G > A (Arg302Gln) mutation was identified as the casual variant (**A**). The Arg302Gln variant was confirmed using sanger sequencing (in red) and represents one of the highly conserved residues across species. A total of 26 variants have been reported in the PRKAG2 protein so far, shown in black (**B**).

Joint-variant calling using data from all 43 samples from the three families in our study resulted in 102,051 nucleotide variants (Fig. 5A). From these, first we filtered out only the rare variants (MAF < 0.1%) followed by identification of the rare variants predicted to have an impact on protein function and/or have disease relevance. The pathogenic variants that segregated through affected and unaffected family members were filtered after overlaying the disease inheritance pattern observed in each pedigree. We identified a pathogenic autosomal dominant missense p.Arg302Gln mutation in the CBS1 domain of PRKAG2 protein which impacts the AMP/ATP binding pocket (Fig. 5B and Supplementary Figure S1). This PRKAG2 mutation was validated using Sanger sequencing on all the individuals (Supplementary Figure S2 and S3). The mutation showed complete phenotype genotype correlation in all the three families (Table 1 and Supplementary Figure S4).

**Table 1:** PRKAG2 Mutation Status. Results from whole exome sequencing and Sanger sequence validation has been summarized.

Sample ID	Patient #	Sequencing data available	Sanger sequencing	Mutation identified (exome sequencing)	Mutation identified (sanger sequencing)
35832_FGD0128	AIV-1	Yes	Yes	c.905G > A (Arg302Gln)	c.905G > A (Arg302Gln)
35833_FGD0128	AIV-2	Yes	Yes	c.905G > A (Arg302Gln)	c.905G > A (Arg302Gln)
39076_FGD0128	AIV-3	Yes	Yes	c.905G > A (Arg302Gln)	c.905G > A (Arg302Gln)
39064_FGD0128	AIV-4	Yes	Yes	None	None
39065_FGD0128	AIV-6	Yes	Yes	None	None
39062_FGD0128	AIV-7	Yes	Yes	None	None
39061_FGD0128	AIV-8	Yes	Yes	c.905G > A (Arg302Gln)	c.905G > A (Arg302Gln)
39066_FGD0128	AIV-9	Yes	Yes	None	None
39067_FGD0128	AIV-10	Yes	Yes	None	None
39068_FGD0128	AIV-11	Yes	Yes	c.905G > A (Arg302Gln)	c.905G > A (Arg302Gln)
39075_FGD0128	AIV-12	Yes	Yes	None	None
35829_FGD0128	AIV-13	Yes	Yes	None	None
39074_FGD0128	AIV-14	Yes	Yes	None	None
39059_FGD0128	AIV-15	Yes	Yes	None	None
35831_FGD0128	AV-1	Yes	Yes	c.905G > A (Arg302Gln)	c.905G > A (Arg302Gln)
35834_FGD0128	AV-2	Yes	Yes	None	None
39073_FGD0128	AV-3	Yes	Yes	None	None
39063_FGD0128	AV-4	No	Yes	NA	None
39060_FGD0128	AV-5	Yes	Yes	None	None
39077_FGD0128	AV-6	Yes	Yes	None	None
41135_FGD0128	AV-7	Yes	Yes	None	None
39078_FGD0128	AV-8	Yes	Yes	None	None
41133_FGD0128	AV-9	Yes	Yes	None	None
41137_FGD0128	AV-11	Yes	Yes	c.905G > A (Arg302Gln)	c.905G > A (Arg302Gln)
41136_FGD0128	AV-10	Yes	Yes	None	None
41134_FGD0128	AV-12	Yes	Yes	None	None
35830_FGD0128	AV-13	Yes	Yes	None	None
41289_FGD0137	BII-2	Yes	Yes	c.905G > A (Arg302Gln)	c.905G > A (Arg302Gln)
77259_FGD0137	BIII-1	Yes	Yes	c.905G > A (Arg302Gln)	c.905G > A (Arg302Gln)
80726_FGD0137	BIII-3	Yes	Yes	c.905G > A (Arg302Gln)	c.905G > A (Arg302Gln)
41288_FGD0137	BIII-4	Yes	Yes	c.905G > A (Arg302Gln)	c.905G > A (Arg302Gln)
98267_FGD0137	BIII-5	Yes	Yes	c.905G > A (Arg302Gln)	c.905G > A (Arg302Gln)
77260_FGD0137	BIV-1	Yes	Yes	None	None
80721_FGD0137	BIV-2	Yes	Yes	c.905G > A (Arg302Gln)	c.905G > A (Arg302Gln)
97442_FGD0137	BIV-4	Yes	Yes	None	None
57108_FGD0314	CII-1	Yes	Yes	None	None
57116_FGD0314	CII-2	Yes	Yes	None	None
57114_FGD0314	CIII-1	Yes	Yes	None	None
57112_FGD0314	CIII-2	Yes	Yes	None	None
57110_FGD0314	CIII-3	Yes	Yes	None	None
57113_FGD0314	CIII-5	Yes	Yes	c.905G > A (Arg302Gln)	c.905G > A (Arg302Gln)
57107_FGD0314	CIII-6	Yes	Yes	None	None
57111_FGD0314	CIV-1	Yes	Yes	c.905G > A (Arg302Gln)	c.905G > A (Arg302Gln)
57115_FGD0314	CIV-2	Yes	Yes	c.905G > A (Arg302Gln)	c.905G > A (Arg302Gln)

**Table 2:** Cardiac magnetic resonance imaging (CMRI) findings in the selected individuals, LVEF: Left ventricular ejection fraction, RVEF: Right ventricular ejection fraction, LGE: late gadolinium enhancement.

Patient #	AIV-8	AIV-11	AV-1	AV-11	BIV-2	CIII-5	CIV-1	CIV-2
**Left ventricle**								
End-diastolic volume (mL)	167	152	77	69	210	149	109	101
End-systolic volume (mL)	70	69	22	30	107	61	45	39
Maximum septal thickness (mm)	14	18	11	6	30	29	13	10
Maximum lateral wall thickness (mm)	13	12	8	5	17	18	8	9
Stroke volume (mL)	97	83	55	42	103	88	64	61
Cardiac output (L/min)	5.8	5.5	5.4	3.7	5.4	4.6	5.4	4.7
LVEF (%)	58	55	70	65	49	59	59	61
Mass (gm)	154	134	63	36	377	283	79	63
LV CMRI LGE (%)	Absent	Absent	Absent	Absent	9.7	10	Absent	Absent
**Right ventricle**								
End-diastolic volume (mL)	161	139	77	57	170	123	113	101
End-systolic volume (mL)	68	56	25	16	65	39	51	39
RV free wall thickness (mm)	7	7.5	2	3	11	9	6	2
Stroke volume (mL)	94	83	52	41	104	84	63	62
RVEF (%)	58	60	68	72	62	68	55	62
RV CMRI LGE (%)	Absent	Absent	Absent	Absent	Absent	Absent	Absent	Absent

### Kinship analysis

**Figure 6:**
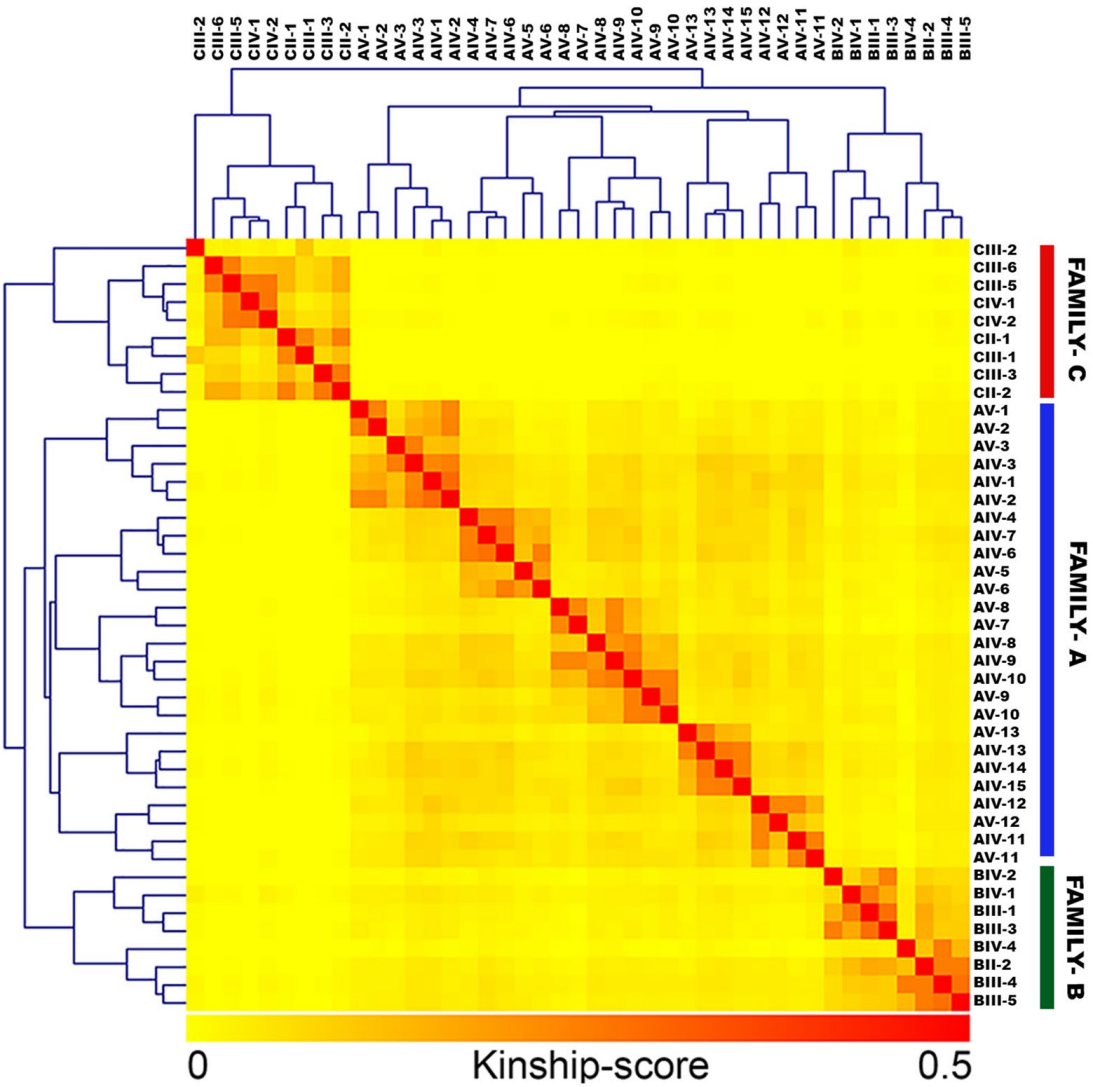
Kinship analysis of the 3 families showed that the families are completely unrelated and have no founder effects. Genotype of each individual was compared with all the other individuals. Kinship was scored on a scale of 0–0.5, where kinship score 0.5 represents the identical individual and 0 represents the completely unrelated individual.

All the three families investigated in this study belonged to the same geographical region in India. Since all affected members in the three families showed the same pathogenic variant in the PRKAG2 gene, we performed an unbiased relatedness analysis across all the three families to check if all the families are coming from the same founder population. The sample identity was computed based on the high-confidence set of single nucleotide variants (SNVs) that passed GATK hard-filtering criteria. The three families did not show any relatedness among them and formed three distinct clusters (Fig. 6) in a hierarchical cluster analysis of the pair-wise kinship matrix.

### Clinical course

At cardiac diagnosis, this 22-patient cohort had symptoms ranging from dyspnea on exertion (NYHA class I–II), palpitations, syncope and pre-syncope. Over the 7.0 ± 1.5 years follow up period, 11 patients (11/22; 50%) developed adverse cardiovascular events. Six patients (AII-1, AIII-1, AIII-3, AIII-5, BII-1 and CII-4) had sudden cardiac deaths (6/22; 27%). The mean age of the 6 patients who succumbed to sudden cardiac death was 56.3 ± 4 (SD) years. Four patients out of the 6 sudden cardiac deaths (4/6; 66%) had documented electrocardiographic evidence of conduction system disease (2 patients with high grade atrio-ventricular block, 1 patient with Mobitz Type 2 atrio-ventricular block, 1 patient with complete heart block), while we did not have data pertaining to the remaining two patients. Only one patient, among the 6 sudden cardiac deaths had received a permanent pacemaker implant for Mobitz type II atrio-ventricular block.

Eight patients required a permanent pacemaker implant for conduction system disease (8/22; 36%). The mean age of the eight patients who required permanent pacing therapy was 43.4 ± 16 (SD) years. Of the eight patients who received permanent pacing, 5 patients had atrio-ventricular conduction system disease, 2 patients had severe sinus node dysfunction, one patient had both atrio-ventricular as well as sinus node dysfunction with evidence of atrio-ventricular re-entrant tachycardia (AVRT). The youngest patient to receive a pacemaker implant was an 11-year-old boy with Mobitz type II atrio-ventricular block along with new onset right bundle branch block (RBBB).

Three patients (3/22;14%) had documented atrial fibrillation and atrial flutter. Two patients had AVRT (2/22;9%). One of these two patients had rapid antidromic AVRT along with high grade atrio-ventricular AV block and underwent radiofrequency ablation of his accessory pathway after having had a permanent pacemaker implant at the age of 49 years, while the other patient was found to have orthodromic AVRT and he underwent radiofrequency ablation of the accessory pathway at the age of 26 years. One patient (BIII-1) developed a hemorrhagic stroke (right parieto—occipital bleed with left sided hemiparesis and a left thalamic bleed) but did not exhibit evidence of cardiac arrhythmias on ambient electrocardiographic monitoring.

Over the follow up period, 11 patients (11/22; 50%) developed progressive exertional dyspnoea by at least one NYHA functional class grade. 15 patients (15/22; 68%) were noted to have impaired left ventricular ejection fraction (LVEF) at the end of the follow up period (LVEF 40–58%; Mean LVEF 49.2 ± 4.3 (SD)%). During follow up, progressive deterioration of the left ventricular ejection fraction (drop in EF by > 10% and to EF < 50%) was observed in 10 patients (10/22; 45%; Mean LVEF 48 ± 3.7 (SD)%). This degree of left ventricular ejection fraction impairment in these 10 patients were observed as early as the third decade of life (age range: 30–59 years; Mean 46.5 ± 3.6 (SD) years). The mean left ventricular ejection fraction of the entire cohort (N = 22) was 53 ± 6.8 (SD)%. The diastolic function was impaired in 19 patients at the end of the follow-up period. (19/22; 86%).

The key findings of each patient related to the clinical course have been summarized in Supplementary Table S1a, S1b and S1c.

## Discussion

PRKAG2 cardiomyopathy is a rare, early onset autosomal dominant cardiomyopathy with progressive clinical deterioration with worsening left ventricular function and heart failure, premature and severe conduction system disease necessitating permanent cardiac pacing therapy and sudden cardiac death^5,8,15–19^. The prevalence of the PRKAG2 syndrome is currently unknown, however, as more genetic testing is incorporated into the work up of patients with HCM, the observed prevalence may be rising. Arad et al. reports that genetically confirmed PRKAG2 syndrome was found in 29% (7/24 patients) among a sub-group of patients with LVH and ventricular pre-excitation^4^.

In this study we investigated the three unrelated index patients (and their respective family members) who presented with HCM like clinical phenotype. Detailed clinical and genetic evaluation revealed PRKAG2 cardiomyopathy in 22 members in these three families. The clinical course of these 22 patients was followed up for a period of 7.0 ± 1.5 years. The mean age of our patients at diagnosis was 39.5 ± 18 yrs and is consistent with age distributions described in earlier studies^6,17,20–22^.

To ascertain our clinical diagnosis, whole exome sequencing was performed to identify the causal variant for the disease in these families. We identified the pathogenic missense variant in the PRKAG2 gene, p.Arg302Gln as the causal variant in this cohort. The remaining 28 clinically unaffected members were negative for PRKAG2 or any other HCM related gene mutations. Arg302Gln mutation has been shown to aberrantly activate AMP Kinase leading to glycogen accumulation in the cardiac muscles^23^.

PRKAG2 p.Arg302Gln is one of the most common mutations identified with this rare cardiomyopathy followed by p.Asn488Ile (57% and 21% respectively)^18^. Three new mutations in patients with the PRKAG2 syndrome were recently reported in a French cohort of 34 patients (p.Ser333Pro, p.Val336Ala and p.His530Arg)^16^. This recent French cohort demonstrated that the majority of patients carried the Arg302Gln missense variant, underlining the fact that this is a relatively frequent variant in this disease. A novel PRKAG2 missense mutation(p.Val336Leu) was also reported in a three generation Chinese family by Zhang et al.^24^. Sternick et. Al. described a cohort of 10 PRKAG2 cardiomyopathy patients and all of them possessed the p.Arg302Gln missense variant^25^. A very recent PRKAG2 cohort described two novel variants, Leu341Ser and Lys485Glu, which were associated with a severe cardiac phenotype^26^ (Fig. 5B).

This PRKAG2 cardiomyopathy cohort demonstrated a clinical profile dominated by cardiac manifestations, including biventricular hypertrophy (19/22 patients with left and right ventricular hypertrophy), without evidence of skeletal myopathy as seen in patients with the PRKAG2 syndrome with the Asn488Ile mutation^27^. Three patients, AV-1, AV-11 and CIV-2 aged 9, 7 and 13 years of age respectively, were heterozygous for the pathogenic missense variant, but did not demonstrate LVH. LVH is the commonest cardiac manifestation of PRKAG2 cardiomyopathy and is often concentric in distribution, predominantly involving the septum or the inferolateral wall^28^. Our patients had a predominantly concentric pattern of LVH. An apical distribution of LVH in PRKAG2 patients was described for the first time by Hu et. al. recently^26^. None of our patients had left ventricular mid-cavitary or outflow tract obstruction. Endomyocardial biopsy findings of patient CIII-5 included a combination of histopathologic features consistent with a storage disease, i.e., abundantly vacuolated myocytes with magenta positive granules with Per-iodic acid Schiff stain, indicating glycogen deposition. Notably, myofibrillar disarray, which is commonly seen in HCM patients of the sarcomeric type was very minimal. The LGE burden (indicative of fibrotic scarring) was minimal in the 8 patients who underwent CMR.

Recent studies indicate that PRKAG2 cardiomyopathy has a high incidence of cardiac conduction system disease with high pacemaker implantation^18^ and sudden cardiac death (SCD) rates^16^. Over a 7.0 ± 1.5 years follow up period, our cohort showed progressive conduction system disease with 36% (8 of 22) needing permanent cardiac pacing for atrio-ventricular blocks and sinus node dysfunction along with 27% (6 of 22) succumbing to SCD.

Ventricular pre-excitation pattern (WPW pattern) was seen in 77.2% (17 of 22), which is the most common electrocardiographic feature of patients with the PRKAG2 syndrome^6,19,20,25,27^. Two patients (BIII-4 and BIV-2) had presented with rapid AVRT (accessory pathway) and had to undergo radio-frequency modification of the accessory pathways. It is conceivable that ventricular fibrillation, secondary to a supraventricular tachycardia conducting rapidly through accessory pathways, may be a mechanism of sudden cardiac death in patients with the PRKAG2 disease, in addition to an abrupt advanced atrio-ventricular or sino-atrial block leading to asystole^20,25,27,29^.

77% of our patients had the WPW pattern, which is associated with accessory pathways (short PR interval with delta wave), and is the electrocardiographic hallmark of the PRKAG2 syndrome^6,27,30^. The role of PRKAG2 in cardiac development and annulus fibrosus integrity may contribute to these accessory pathways^17,25,30^. Radiofrequency ablation in these accessory bypass pathways may lead to iatrogenic atrio-ventricular blocks^27^. Patient BIII-4 had an antidromic AVRT and electrophysiological study which showed an atrio-fascicular pathway as the only source of atrio-ventricular conduction with distal attachment close to the His bundle. Patient underwent dual chamber permanent pacemaker implantation for documented atrioventricular block and subsequent radio-frequency ablation of the accessory pathway. The p.Arg302Gln mutation identified in our cohort, has a higher prevalence of pre-excitation pattern on ECG, syncope and rate of pacemaker implantation as compared to patients with the p.Asn488Ile mutation^18^.

None of the patients in our cohort had received an Automated Implantable Cardioverter Defibrillator (AICD) for the prevention of SCD. AICD implants have been reported for both primary as well as secondary prevention of SCD in PRKAG2 patients^20,25,27^. Six patients (6/22; 27%) in our cohort had succumbed to sudden cardiac death. Two patients among them ( 2/6; AII-1 and AIII-1) did not have documented conduction system disease or symptoms suggestive of conduction disease (syncope or pre-syncope) prior to their SCD events and their SCD risk stratification based on established sarcomeric HCM risk factors had not qualified them for an AICD or pacemaker implant. The remaining four patients (AIII-3, AIII-5, BII-1 and CII-4) had been detected to have evidence of advanced atrio-ventricular blocks and had symptoms attributable to it. These four patients had been advised permanent pacing therapy, of which only one patient underwent the procedure. Ideally, a dual chamber AICD would have been the preferred mode of permanent pacing therapy in our patients with the morphological substrate of LVH and conduction system disease. However, there were two important considerations which led us to choose a conventional dual chamber pacing system over an AICD in the eight patients who required pacing therapy. First, none of the eight patients who had required a pacemaker had documented ventricular tachycardia or ventricular fibrillation during their clinical course prior to the device implant (pacing indications were atrio-ventricular blocks and sinus node disease). Second, and equally important, an AICD is considerably more expensive than a conventional dual chamber pacing system, especially in the context of the socio-economically disadvantaged background to which our patients belonged. These considerations led us to conclude that a conventional dual chamber pacing system would be the most cost-effective mode of delivering permanent pacing therapy in our patients.

According to the limited literature available, SCD occurs in about 10% of patients^20,25,27,29^. The selection of those patients who would benefit from AICD therapy for primary prevention is not clear, although a strategy based on evaluation of individual risk factors should probably be considered, as is the established practice for HCM of the sarcomeric type^29^. Similar to this study, there was a high incidence of sudden cardiac death in the recent French (32%) and European (13%) cohorts^16,31^ which clearly exceed the event rates noted in high risk sarcomeric HCM cohorts. Because of the paucity of follow up data published regarding this rare disease, risk stratification for ventricular arrythmias and sudden cardiac death remains challenging. The identification of those PRKAG2 cardiomyopathy patients who could benefit from AICD therapy for primary prevention is still unclear. Based on our evolving understanding of the natural history of this rare disease, we propose that risk factors such as syncope of probable arrhythmic origin, family history of SCD or pacemaker implantation, extent of LVH, documented non-sustained ventricular tachycardia and extent of LGE on cardiac MRI must be evaluated. In addition, an electrophysiological study (EPS) may aid risk stratification, especially in the presence of patterns of ventricular pre-excitation with rapid SVT and atrioventricular conduction disease. Adopting such a combined approach, the phenotypic expression of PRKAG2 cardiomyopathy alone would certainly lower the threshold to initiate the discussion of AICD implantation for primary prevention with the patient. Larger follow up studies and data from PRKAG2 cardiomyopathy patients with implanted devices will throw further light on this risk stratification process.

There was progressive deterioration in the left ventricular systolic function. The mean LVEF of the 22 patients at the end of the follow up period was 53 ± 7% (40–65). Three patients (3/22; 14%) in our cohort were asymptomatic. Eleven patients (11 of 22; 50%) had progressive worsening of effort dyspnea by at least one NYHA functional class at the end of the follow up period. Systolic heart failure has been reported in approximately 12% of patients previously^18^. All the patients in our study were largely asymptomatic up until the second decade of life. This was consistent with the earlier published data that the onset of symptoms in the PRKAG2 syndrome commonly occurs within the first three decades of life and the earliest symptoms are often due to arrhythmias and heart failure (bradyarrhythmias or tachyarrhythmias)^6,20,32^,. Cardiac transplants for end stage heart disease have been reported infrequently in published literature.

A severe form of PRKAG2 disease has been reported in patients with the p.Arg531Gln mutation, characterized by an early onset and rapid progression of disease leading to death within the first 3 months of life^33^. The p.Glu506Lys mutation initially described in a Turkish family with the PRKAG2 cardiac syndrome, was associated with a progressive HCM leading to heart failure with reduced ejection fraction at ~ 40 years of age^34^. Similarly, patients with the p.His530Arg mutation in a French cohort, manifested with an early and severe evolution to cardiac failure^16^. The novel variants Leu341Ser and Lys485Glu were described very recently by Hu et al. in two individuals and both of them underwent heart transplantation at young ages due to rapidly progressive heart failure^26^. However, the frequency of heart transplantation appears to be much lower in patients with the Arg302Gln and Asn488Ile variants^22^.

The existing literature on PRKAG2 cardiac syndrome had been based on four studies which revealed that 25.8% of the patients were asymptomatic, 75% developed ventricular pre-excitation, 66.8% had HCM and 37.4% needed permanent pacing therapy for atrio-ventricular blocks^17,32,35^. The French cohort, of which the majority were patients carrying the Arg302Gln missense variant, showed that 12% of patients were asymptomatic, 60% had HCM, 77% developed ventricular pre-excitation, 33% developed atrioventricular blocks and 29% required device implantation (5 pacemakers and 5 AICD)^16^.

A recent multicenter, retrospective, longitudinal cohort study of 90 PRKAG2 syndrome patients from 27 European centers was described by Lopez-Sainz et al.^31^. Notably, 88 patients were Caucasians of European ancestry. The 2 non-Caucasian patients were two males from India and Pakistan, respectively. In contrast, our cohort is made up entirely of patients with South Asian ancestry. Interestingly, of the 2 non-Caucasian patients in this European cohort, the patient from Pakistan who had undergone a pacemaker implantation carried the p.His401Asp PRKAG2 variant and the Indian patient carried the p.Leu352Lysfs * 6 frameshift variant. These variants were not observed in our cohort and all of our patients were noted to have the p.Arg302Gln missense variant. Notably, the p.Arg302Gln missense variant identified in our South Asian cohort was present in 32 patients of the European cohort (32/90; 35%) and all of these 32 individuals were from 6 different European countries (Spain, Italy, Israel, Denmark, Portugal, and the United Kingdom). This European cohort had a median age of 40 years (IQR: 19 to 54 years), which was similar to our cohort (Mean 39.5 ± 18.1 years). The patients in our cohort had a higher mean left ventricular maximal wall thickness (Mean 22.9 ± 8.7 mm; range 6–33 mm) when compared to the European cohort (17 ± 7 mm), indicating a milder degree of LVH in the European group. None had evidence of left ventricular outflow tract obstruction > 30 mmHg in both the European as well as our cohort. The mean LVEF in our cohort was 53 ± 7% when compared to the mean LVEF 59 ± 13% in the European study. The natural history of the European cohort revealed that 76% were symptomatic, 71% had HCM, 37% demonstrated ventricular pre-excitation, 53% had evidence of atrio-ventricular blocks, 13% succumbed to sudden cardiac death, 35% needed permanent pacemaker implantation and 4% required cardiac transplantation. Patients with the p.Arg302Gln and p.Asn488Ile variants in the European study exhibited ventricular pre-excitation more frequently.

A clinical comparison between these three previously published groups and our cohort (South Asian Cohort) is summarized in Table 3.

**Table 3:** Comparative analysis of clinical features with other published cohorts.

Clinical manifestations	Single-center South Asian cohort^d^ (N = 22) (%)	Grouped study (4 studies)^a^ (N = 156) (%)	Multi-center French cohort^b^ (N = 34) (%)	Multi-center European cohort^c^ (N = 90) (%)
Symptomatic patients	86	74	77	76
HCM	86	67	60	71
Ventricular pre-excitation	77	75	77	37
Atrioventricular blocks	45	30	33	53
Sudden cardiac death	27	12	32	13
Permanent pacemaker implant	32	43	15	35

^a^Fabris, E. et al., JACC (2013)^17^, Arad, M. et al., JCI (2002)^32^, Zhang, L.-P. et al., J. Electrocardiol (2011).

^b^Thevenon, J. et al., Europace (2017).

^c^Lopez-Sainz et al., JACC (2020).

^d^Current study.

Danon’s disease, Pompe disease, Anderson—Fabry disease and mitochondrial disorders are other important HCM phenocopies which can mimic the PRKAG2 syndrome. A definitive diagnosis of the PRKAG2 syndrome can only be made by genetic testing which identifies a PRKAG2 mutation in a patient with the recognized clinical red flags of this disease.

Molecular testing to confirm the diagnosis of PRKAG2 cardiomyopathy and familial screening of at-risk relatives should be a part of the overall management strategy. We believe that this investigation and extended clinical follow up of this PRKAG2 cohort has revealed invaluable insights into this rare disease. To our knowledge, this study is the first comprehensive analysis of the clinical spectrum, outcomes and genetic analysis of a PRKAG2 cohort from the South Asian region (Indian Subcontinent), and hence, adds a new dimension to understanding this rare cardiomyopathy in terms of clinical data from an entirely different demographic and population perspective.

## Supplementary information

Supplementary Information.
