# Supplementary material for: Phenotypic expression and clinical outcomes in a South Asian PRKAG2 cardiomyopathy cohort
Source: Sci Rep. 2020 Nov 26;10:20610. doi: 10.1038/s41598-020-77124-9 (PMC7691361; doi:10.1038/s41598-020-77124-9)
Supplement: Supplementary file 1 — Supplementary Information. [file 41598_2020_77124_MOESM1_ESM.pdf]

Phenotypic Expression and Clinical Outcomes in a South Asian PRKAG2 Cardiomyopathy Cohort.

Hisham Ahamed[1, #], Aniketh Vijay Balegadde[1], Shilpa Menon[1], Ramesh Menon[2], Aishwarya Ramachandran[1], Navin Mathew[1], K. U. Natarajan[1], Indu Ramachandran Nair[1], Rajesh Kannan[1], Meghna Shankar[2], Oommen K. Mathew[3], Thong T. Nguyen[4], Ravi Gupta[2], Eric W. Stawiski[5], Ramprasad VL[2], Somasekar Seshagiri[4,6] and Sameer Phalke[2, 7, #]

1. Amrita Institute of Medical Sciences and Research, Kochi, India
2. MedGenome Labs, Bangalore, India
3. AgriGenome Labs, Kochi, India
4. Genentech Inc., South San Francisco, USA
5. MedGenome Inc., Foster City, USA
6. SciGenom Research Foundation, Kochi, India
7. SciGenom Labs Pvt Ltd, Kochi, India

# Corresponding Authors:

Hisham Ahamed: ahamed.hisham@gmail.com
Clinical Associate Professor, Amrita Institute of Medical Sciences and Research, Kochi, India

Sameer Phalke: sameer.p@scigenom.com
Senior Scientist, SciGenom Labs Pvt Ltd, Kochi, India

Figure Legend

Supplementary Figure S1: A close up of the AMP binding pocket of WT and PRKAG2 R302Q protein. Substitution of Arginine with Glutamine residue impacts the interactions between amino acids at the AMP binding pocket making AMP more flexible as compared to the WT protein.

Supplementary Figure S2: Verification by sanger sequencing of each of the individual for PRKAG2 mutation in Family A

Supplementary Figure S3: Verification by sanger sequencing of each of the individual for PRKAG2 mutation in Family B (A) and Family C (B)

Supplementary Figure S4: Pedigree of the three families showing segregation of the variant. (A) Family A (FGD0128), (B) Family B (FGD0137) and (C) Family C (FGD0314). Individuals for whom whole exome sequencing was performed are highlighted with asterisk (*). Square represents the males and the circles represent the females. Filled squares and the circles represent the affected individuals. Square or circles with question mark (?) represent the individuals with unknown phenotype. SCD represent the individual who had sudden cardiac death and the Pacemaker represent the individuals who had to get pacemaker. +/+ represent the individuals carrying wild type PRKAG2 allele and +/- represent the individuals carrying heterozygous PRKAG2 mutant allele.

Table Legend

Supplementary Table S1a, S1b and S1c: Clinical evaluation of 50 members from three unrelated families. += Present; 0 = Absent; ST ↑ = ST elevation; α- Right parieto – occipital bleed with left sided hemiparesis in 2017 and a left thalamic bleed in 2018; ß- Documented antidromic AVRT at 200 beats per minute. Electrophysiological study showed atrio-fascicular pathway as only source of atrio-ventricular conduction with distal attachment close to the His bundle. Patient underwent dual chamber permanent pacemaker implantation. Upon recurrence of

AVRT, underwent radio-frequency ablation. µ- Documented orthodromic AVRT (left lateral accessory pathway). Underwent radio-frequency ablation. T

Supplementary Table S2: Data quality and processing summary

Supplementary Table S3a and S3b: Measurement of septal, lateral wall and Right ventricle walls as identified by echocardiography. IVS – Interventricular septum; LW – Lateral wall; RV – Right ventricle

Supplementary Figure S1

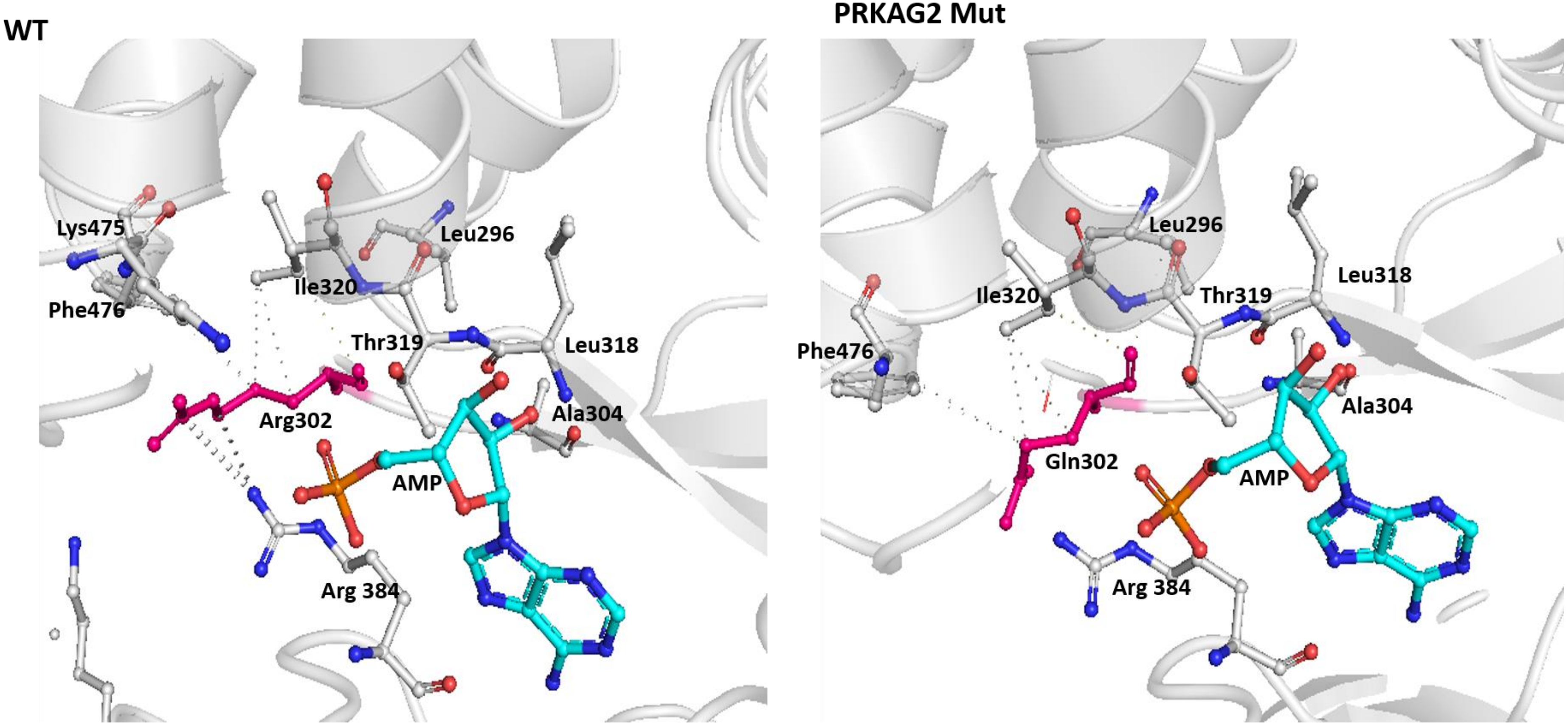

Supplementary Figure S2

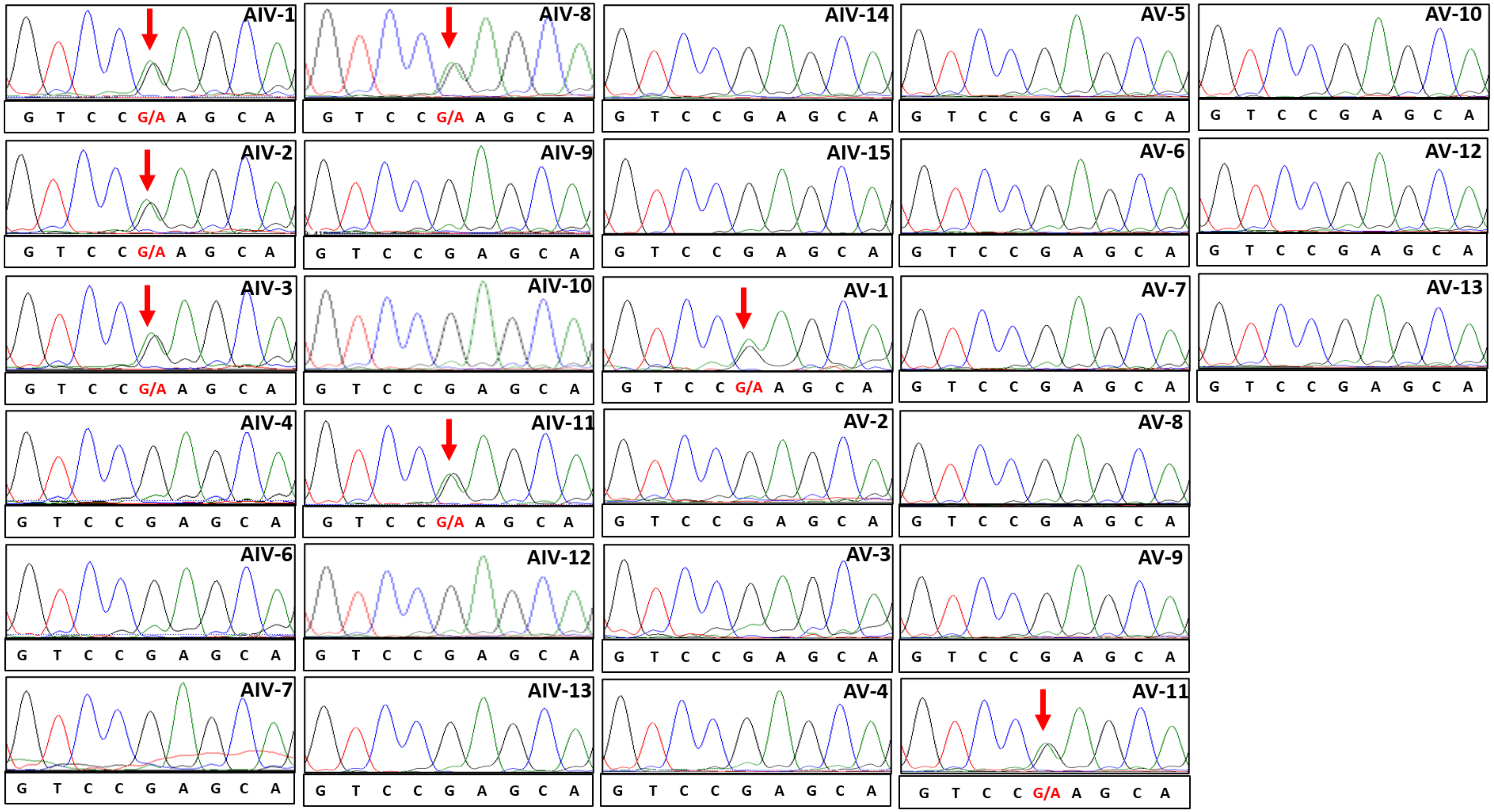

Supplementary Figure S3

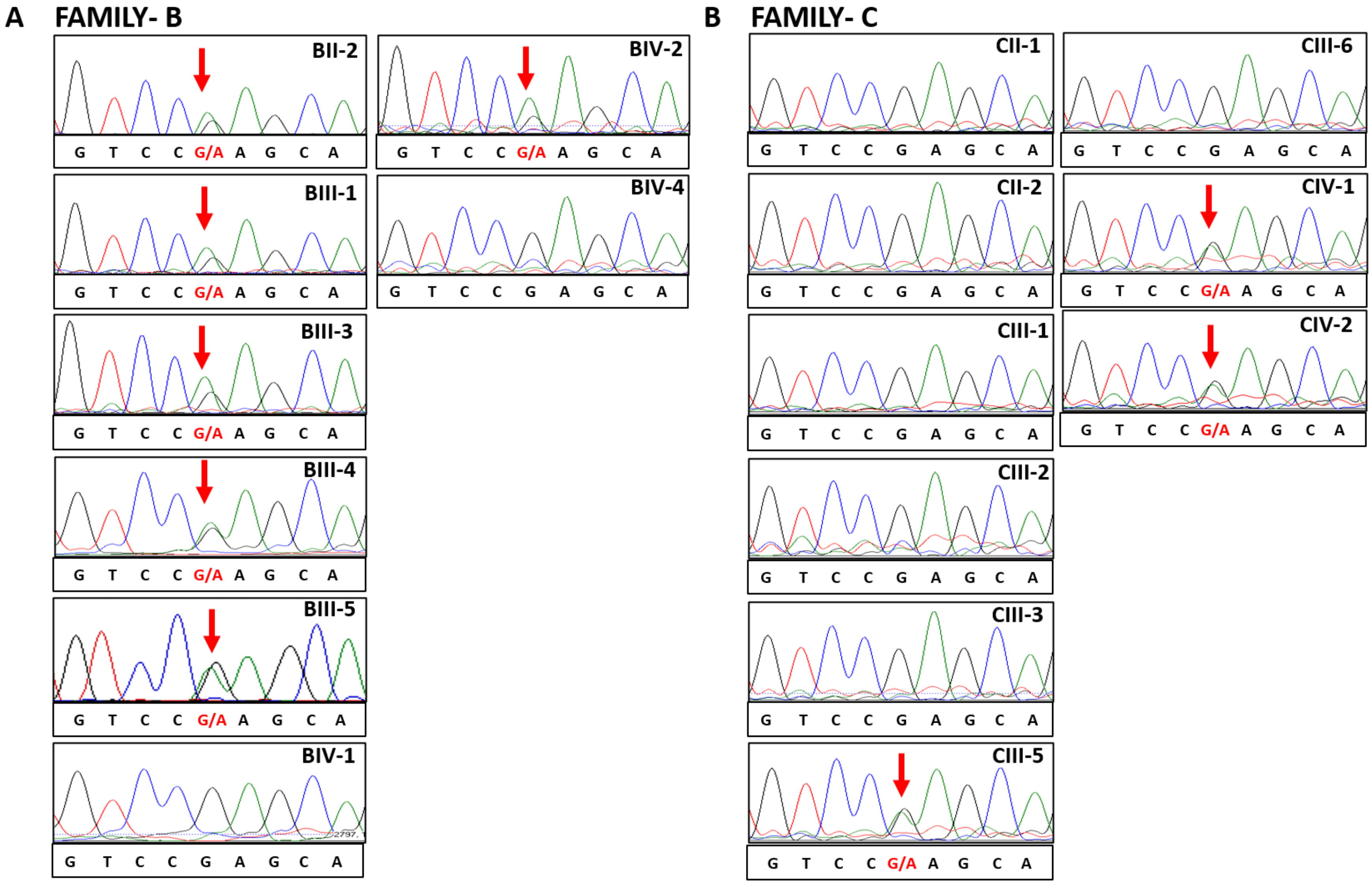

Supplementary Figure S4

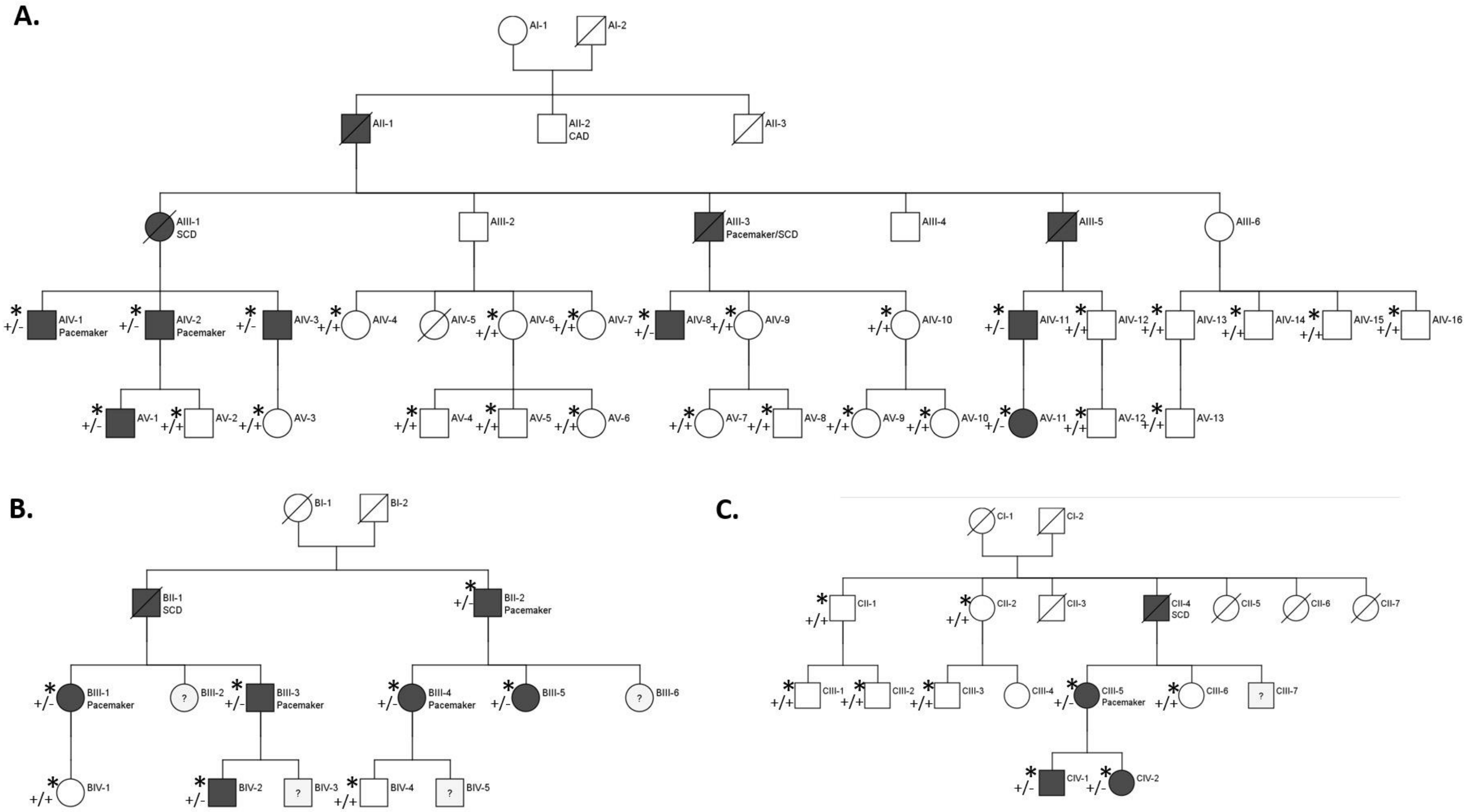

Supplementary Table S1a

Patient #	Sample ID	Age (yrs)	Gender	Presentation	NYHA functional class		AF/Flutter/AVRT	AV Blocks	Sinus node dysfunction
					Initial	Most Recent			
AII-1	#N/A	52	Male	Dyspnea on exertion	II	III	NA	NA	NA
AIII-1	#N/A	52	Female	Dyspnea on exertion	II	II	NA	NA	NA
AIII-3	#N/A	56	Male	Syncope	II	II	0	Mobitz Type 2 (2:1 AV block)	0
AIII-5	#N/A	58	Male	Dyspnea on exertion	II	III	0	High grade AV block	0
AIV-1	35832_FGD0128	32	Male	Syncope	I	II	Atrial flutter	Complete heart block	0
AIV-2	35833_FGD0128	38	Male	Syncope	I	II	Atrial fibrillation	Complete heart block	0
AIV-3	39076_FGD0128	32	Male	Palpitations	I	II	Paroxysmal atrial flutter	0	0
AIV-8	39061_FGD0128	32	Male	Dyspnea on exertion	II	II	0	0	0
AIV-11	39068_FGD0128	42	Male	Dyspnea on exertion	II	II	0	0	0
AV-1	35831_FGD0128	9	Male	Family history	I	I	0	0	0
AV-11	41137_FGD0128	7	Female	Family history	I	I	0	0	0
BII-1	#N/A	56	Male	Syncope	I	II	NA	Complete heart block	0
BII-2	41289_FGD0137	62	Male	Syncope	I	III	0	0	+

BIII-1	77259_FGD0137	62	Female	Syncope	I	II-III	0	Mobitz Type 2; (2:1 AV block)	0
BIII-3	80726_FGD0137	49	Male	Palpitations	II	III	Antidromic AVRT	High grade AV block	+
BIII-4	41288_FGD0137	40	Female	Syncope, Palpitations	II	II	0	0	+
BIII-5	98267_FGD0137	36	Female	Dyspnea on exertion	II	II	0	0	0
BIV-2	80721_FGD0137	26	Male	Palpitations	II	II	Orthodromic AVRT	0	0
CII-4	#N/A	64	Male	Syncope	II	II	NA	High grade AV block	0
CIII-5	57113_FGD0314	35	Female	Dyspnea on exertion	II	III	0	Mobitz Type2 (2:1 AV block)	0
CIV-1	57111_FGD0314	11	Male	Dyspnea on exertion	II	II-III	0	Mobitz Type 2 (2:1 AV block)	0
CIV-2	57115_FGD0314	13	Female	Family screening	I	I	0	0	

Supplementary Table S1b

Patient #	Sample ID	Electrocardiogram				LV Outflow gradient(mm)	Maximum LV wall thickness (mm)	Maximum RV Free wall thickness (mm)	Ejection Fraction (%)
		WPW	Maximum Voltage(mm)	PR interval(ms)	Other				
AII-1	#N/A	+	36	100	T inversions	0	29	7	50
AIII-1	#N/A	+	32	110	T inversions	0	33	6.5	58
AIII-3	#N/A	+	28	108	T inversions	0	30	7.5	45
AIII-5	#N/A	+	29	90	T inversions, IVCD	0	31	7	55
AIV-1	35832_FGD0128	+	40	100	T inversions	0	29	7	50
AIV-2	35833_FGD0128	+	35	90	T inversions	0	32	7.5	40
AIV-3	39076_FGD0128	+	25	110	rSR' in III, IVCD	0	16	7	60
AIV-8	39061_FGD0128	+	29	120	T inversions	0	13	7	60
AIV-11	39068_FGD0128	+	26	100	T inversions, ST elevation V2-V4, rSr' in V2	0	18	7.7	60
AV-1	35831_FGD0128	Absent	28	118	-	0	8	2	65
AV-11	41137_FGD0128	Absent	30	120	-	0	6	3	65
BII-1	#N/A	+	31	100	T inversions	0	30	7.5	52
BII-2	41289_FGD0137	+	42	90	T inversions	0	25	6	47

BIII-1	77259_FGD0137	Absent	25	170	T inversions	0	25	7	52
BIII-3	80726_FGD0137	+	50	80	T inversions, IVCD	0	23	11	50
BIII-4	41288_FGD0137	+	45	90	T inversions, IVCD	0	30	8	49
BIII-5	98267_FGD0137	+	34	100	T inversions	0	16	7	50
BIV-2	80721_FGD0137	+	70	90	T inversions, IVCD	0	30	11	51
CII-4	#N/A	+	30	110	T inversions	0	29	7	45
CIII-5	57113_FGD0314	+	45	100	T inversions	0	29	9	45
CIV-1	57111_FGD0314	Absent	25	90	Biphasic T in V2-V4, IVCD,ST ↑V2,V3,RBBB	0	13	6	62
CIV-2	57115_FGD0314	Absent	25	120	-	0	10	2	60

Supplementary Table S1c

Patient #	Sample ID	LV cavity(End-Diastole), (mm)	Left atrium (mm)	Mitral regurgitation	24 Hr Holter ECG	Permanent Pacemaker	EPS + RFA	AICD	Stroke	Clinical Status
AII-1	#N/A	48	45	Trivial	NA	0	0	0	0	SCD
AIII-1	#N/A	46	48	Trivial	NA	0	0	0	0	SCD
AIII-3	#N/A	50	46	Trivial	NA	+ (Dual Chamber)	0	0	0	SCD
AIII-5	#N/A	52	49	Mild	NA	0	0	0	0	SCD
AIV-1	35832_FGD0128	38	40	Trivial	NA	+ (Dual Chamber)	0	0	0	Alive
AIV-2	35833_FGD0128	40	42	Trivial	NA	+ (Dual Chamber)	0	0	0	Alive
AIV-3	39076_FGD0128	49	41	0	Atrial flutter	0	0	0	0	Alive
AIV-8	39061_FGD0128	48	36	0	Isolated APCs	0	0	0	0	Alive
AIV-11	39068_FGD0128	45	32	0	Isolated APCs	0	0	0	0	Alive
AV-1	35831_FGD0128	39	32	0		0	0	0	0	Alive
AV-11	41137_FGD0128	33	26	0	Normal	0	0	0	0	Alive
BII-1	#N/A	51	48	Trivial	NA	0	0	0	0	SCD
BII-2	41289_FGD0137	45	36	Mild	Prolonged sinus pauses > 3 sec	+ (Dual Chamber)	0	0	0	Alive

BIII-1	77259_FGD0137	38	40	Trivial	Mobitz Type 2, (2:1 AV block)	+ (Dual Chamber)	0	0	Intracranial bleed[α]	Alive
BIII-3	80726_FGD0137	48	35	Trivial	AVRT/High grade AV block/SSS	+(Dual Chamber)	+ ß	0	0	Alive
BIII-4	41288_FGD0137	38	42	Trivial	Sinus pauses>3 sec Frequent APCs	+(Dual Chamber)	0	0	0	Alive
BIII-5	98267_FGD0137	48	33	0	Isolated APCs	0	0	0	0	Alive
BIV-2	80721_FGD0137	40	42	0	NA	0	+µ	0	0	Alive
CII-4	#N/A	50	44	Trivial	NA	0	0	0	0	SCD
CIII-5	57113_FGD0314	34	36	Trivial	Intermittent Mobitz type 2 (2:1 AV block)	+(Dual Chamber)	0	0	0	Alive
CIV-1	57111_FGD0314	40	29	0	Mobitz type 2 (2:1 AV block)	Awaiting pacemaker	0	0	0	Alive
CIV-2	57115_FGD0314	40	33	0	Isolated APCs	0	0	0	0	0

Supplementary Table S2

Patient #	Sample ID	Total data (Mb)	Raw data >= Q30 (%)	Alignment (%)	Duplicate (%)	On Target (%)	Average Depth (X)
AIV-9	39066_FGD0128	5945.399708	94.12	99.9821	2.2851	94.472	20.06413225
AV-3	39073_FGD0128	7462.381042	94.105	99.9886	2.6677	94.948	25.51270749
AIV-10	39067_FGD0128	5261.329844	96.025	99.9795	1.6611	78.911	36.51271623
AV-5	39060_FGD0128	5668.765292	96.265	99.9276	8.0158	77.962	37.73076802
AIV-15	39059_FGD0128	6004.302392	96.375	99.9508	9.6263	79.048	41.2397796
AIV-2	35833_FGD0128	5805.624746	96.315	99.9152	9.6746	37.942	41.66271931
AIV-8	39061_FGD0128	5315.197282	93.995	99.9825	3.9699	72.288	42.16513684
AV-6	39077_FGD0128	6659.245262	96.045	99.9868	2.2073	77.543	44.13489176
AIV-6	39065_FGD0128	4872.81379	94.03	99.9806	2.2873	86.162	46.39940887
AIV-11	39068_FGD0128	6942.967316	96.645	99.9865	2.1878	78.601	46.93409383
AIV-7	39062_FGD0128	5993.69283	93.9	99.9291	8.0559	65.262	46.9562903
AV-12	41134_FGD0128	6929.446474	96.305	99.9323	8.4495	82.012	47.22231078
AV-11	41137_FGD0128	6625.187816	96.58	99.9359	7.5409	82.012	47.23040904
AIV-3	39076_FGD0128	5038.183252	92.1	99.9796	1.6484	82.495	48.57302548
AIV-14	39074_FGD0128	5014.52125	92.29	99.9859	2.6541	83.273	48.96437764
AV-7	41135_FGD0128	6842.84284	96.66	99.9459	9.8964	82.012	49.93078661
AIV-4	39064_FGD0128	5194.471574	94.5	99.9247	9.4037	93.678	51.06557524
AV-9	41133_FGD0128	7476.793992	97.065	99.9124	8.374	82.012	54.10422167
AV-10	41136_FGD0128	8103.826626	96.535	99.923	8.165	82.012	54.72111119
AIV-13	35829_FGD0128	5627.763054	94.04	99.9833	2.646	92.415	55.28989374
AIV-12	39075_FGD0128	8290.475008	96.315	99.9335	13.5428	94.081	55.35150737
AIV-1	35832_FGD0128	5901.467768	93.89	99.9814	2.8652	85.883	56.66559602
AV-1	35831_FGD0128	5882.880272	93.96	99.9817	2.986	87.936	56.78203054
AV-13	35830_FGD0128	5812.254854	93.6	99.9833	2.5827	90.93	56.99061477

AV-8	39078_FGD0128	8376.046708	96.065	99.9057	12.6487	112.16	59.34781514
AV-2	35834_FGD0128	6542.363712	93.74	99.9886	3.6447	96.999	63.50805738
CIII-3	57110_FGD0314	7570.12377	95.585	99.9571	6.7673	67.237	74.28865838
CIII-5	57113_FGD0314	7562.504612	95.54	99.9486	8.3693	68.516	74.68226135
CIII-1	57114_FGD0314	7627.88429	94.895	99.9537	6.5901	69.934	76.07704264
BIV-4	97442_FGD0137	7967.910922	94.29	99.9593	6.1302	72.903	78.85203509
BII-2	41289_FGD0137	8236.60304	95.28	99.8984	10.2686	73.636	80.46440433
BIII-1	77259_FGD0137	8492.65374	94.025	99.9444	6.7292	74.543	80.65857583
BIV-1	77260_FGD0137	8366.655716	95.265	99.9466	7.2134	74.076	81.2257712
BIII-4	41288_FGD0137	8200.700374	95.11	99.9557	7.448	74.592	81.35222321
CIII-6	57107_FGD0314	8490.685908	94.83	99.9546	5.6737	75.339	81.7287164
CII-1	57108_FGD0314	8626.886398	95.33	99.9436	6.7414	78.938	85.99279801
CIII-2	57112_FGD0314	8667.843034	95.65	99.9484	7.0791	79.584	86.03016437
CIV-2	57115_FGD0314	9527.421104	94.995	99.9493	6.4334	85.362	92.56521467
BIV-2	80721_FGD0137	9966.067648	95.305	99.9439	8.122	86.13	94.75238126
BIII-3	80726_FGD0137	11051.83436	95.65	99.9346	9.812	92.346	101.6748419
CIV-1	57111_FGD0314	10641.36773	95.395	99.9421	7.8813	94.714	103.2357183
CII-2	57116_FGD0314	15077.97242	95.13	99.9551	5.7418	130.923	141.9861909

Supplementary Table 3a

Patient #	AII-1	AIII-1	AIII-3	AIII-5	AIV-1	AIV-2	AIV-3	AIV-8	AIV-11	AV-1	AV-II
Maximum IVS thickness (mm)	29	33	30	31	29	32	16	13	18	11	6
Maximum LW thickness (mm)	17	18	16	18	17	18	12	13	12	8	5
Maximum RV wall thickness (mm)	7	6.5	7.5	7	7	7	7	7	7.5	2	3

Supplementary Table 3b

Patient #	BII-1	BII-2	BIII-1	BIII-3	BIII-4	BIII-5	BIV-2	CII-4	CIII-5	CIV-1	CIV-2
Maximum IVS thickness (mm)	30	25	25	23	30	16	30	29	29	13	10
Maximum LW thickness (mm)	18	16	18	15	20	12	17	16	18	8	9
Maximum RV wall thickness (mm)	7.5	6	7	11	8	7	11	7	9	6	2
